# Multi-event capture–recapture modeling of host–pathogen dynamics among European rabbit populations exposed to myxoma and Rabbit Hemorrhagic Disease Viruses: common and heterogeneous patterns

**DOI:** 10.1186/1297-9716-45-39

**Published:** 2014-04-05

**Authors:** Simone Santoro, Isa Pacios, Sacramento Moreno, Alejandro Bertó-Moran, Carlos Rouco

**Affiliations:** 1 Department of Wetland Ecology, Doñana Biological Station-CSIC, Américo Vespucio s/n, 41092 Seville, Spain; 2 Ethology and Biodiversity Conservation Department, Doñana Biological Station-CSIC, Américo Vespucio s/n, 41092 Seville, Spain; 3 Wildlife Ecology and Management Team, Landcare Research, PO Box 1930, Dunedin 9054, New Zealand

## Abstract

Host–pathogen epidemiological processes are often unclear due both to their complexity and over-simplistic approaches used to quantify them. We applied a multi-event capture–recapture procedure on two years of data from three rabbit populations to test hypotheses about the effects on survival of, and the dynamics of host immunity to, both myxoma virus and Rabbit Hemorrhagic Disease Virus (MV and RHDV). Although the populations shared the same climatic and management conditions, MV and RHDV dynamics varied greatly among them; MV and RHDV seroprevalences were positively related to density in one population, but RHDV seroprevalence was negatively related to density in another. In addition, (*i*) juvenile survival was most often negatively related to seropositivity, (*ii*) RHDV seropositives never had considerably higher survival, and (*iii*) seroconversion to seropositivity was more likely than the reverse. We suggest seropositivity affects survival depending on trade-offs among antibody protection, immunosuppression and virus lethality. Negative effects of seropositivity might be greater on juveniles due to their immature immune system. Also, while RHDV directly affects survival through the hemorrhagic syndrome, MV lack of direct lethal effects means that interactions influencing survival are likely to be more complex. Multi-event modeling allowed us to quantify patterns of host–pathogen dynamics otherwise difficult to discern. Such an approach offers a promising tool to shed light on causative mechanisms.

## Introduction

Emerging and re-emerging infectious diseases present one of the most pressing issues facing wild vertebrate populations in the 21^st^ century [1]. However, relatively little is yet known about the exposure dynamics of infectious agents in their individual hosts, and what determines their impact on life-history traits such as survival [2]. A comprehensive understanding of the ecological processes influencing pathogen dynamics in natural host populations is of crucial importance to predicting both the dynamics of infectious diseases and the risks that they may entail for animal populations [3,4].

The European wild rabbit (*Oryctolagus cuniculus*) and the two main viral diseases that affect them (myxomatosis, and Rabbit Hemorrhagic Disease) represent an important system for addressing wildlife eco-epidemiological issues. European rabbits are a multifunctional keystone species; not only do they alter plant species composition and vegetation structure, but they also represent the bulk of the diet of a wide variety of Iberian predators [5]. These include the Iberian lynx (*Lynx pardinus*) and the Spanish imperial eagle (*Aquila adalberti*), both seriously threatened [5-7]. The arrival of myxoma virus (MV) and Rabbit Hemorrhagic Disease Virus (RHDV) on the Iberian Peninsula has caused a marked decline in rabbit populations over the last 50 years [8-11], threatening further population losses among their predator species [12,13]. In an attempt to prevent local extinction in its native range, and to avoid predator co-extinctions, the European rabbit has been made a conservation priority in both Spain and Portugal [14-17]. In addition the loss of rabbits, considered a pest species across most of their introduced range [18], has in some areas of the Iberian Peninsula caused large-scale economic loss and environmental degradation (e.g. [19,20]).

In spite of the ecological and economic relevance of these issues, disease surveillance still broadly uses simple counts of infected and uninfected animals, although more accurate statistical tools that account for imperfect detection are now available (see [21] for a review of capture–recapture modeling in epidemiology). A few studies have used simple Cormack–Jolly–Seber (CJS) capture–recapture models to investigate MV and RHDV effects on rabbit population dynamics (e.g. [22,23]). As an extension of single-state (CJS) models, multi-state capture–recapture models allow the direct estimation and testing of hypotheses about seroconversion rates and state-specific survival rates [24-26]. However, assessment of the infectious status of all captured individuals is often unfeasible, and some data rearrangement (e.g. data-censoring) is usually required for the application of multi-state models (reviewed in [27]). To avoid these limitations, multi-event capture–recapture models have been recently developed [28] allowing uncertainty in state assessment to be modeled. Here we apply this novel approach to test hypotheses about the effects on survival of, and the dynamics of host immunity to, both MV and RHDV (which occur naturally in the study area). As a general prediction, based on previous studies, we expected MV and RHDV seropositives to have higher survival rates than seronegatives (e.g. [29-31]). In addition, we aimed to do the following: (*i*) estimate monthly survival rates dependent on antibody status, age (juveniles *vs.* adults) and sex, (*ii*) assess monthly seroconversion rates (from seropositive to seronegative, and vice versa) with respect to both diseases according to hosts’ age and sex, (*iii*) quantify the dynamics of seroprevalence to each virus over time, expressed as the seropositive probability, and (*iv*) examine relationships between population size and seroprevalence.

## Materials and methods

### Ethic statement

All animal manipulations reported in this paper were made in accordance with Spanish and European regulations (Law 32/2007, R.D. 1201/2005 and Council Directive 2010/63/EU).

### Study area and sampling

The study was carried out in the southwestern Iberian Peninsula (Hornachuelos Natural Park; 37°49′ N, 5°15′ W; 100–700 m elevational range), where the climate is Mediterranean with hot, dry summers and cool, wet winters [32]. A rabbit breeding program was implemented in the area in 2008 for conservation purposes; the main objective was to increase rabbit abundance in the area to enhance survival of threatened predators. Three enclosures (E1, E2, E3; about 4 ha each) were built as breeding zones for rabbits, being surrounded by 2.5-m-high chain-link fence to both prevent rabbit dispersal and exclude terrestrial predators [33]. Each enclosure contained 30 regularly distributed artificial warrens, water and pellet food were supplied *ad libitum*, and grass was sown to increase the availability of fresh food.

From May 2008 to April 2010, nine one-day live-trapping sessions were carried out at each enclosure. Time-intervals between capture sessions varied slightly across enclosures and through the time period; on average a capture session was performed every 2.9 months (SE: 0.18). Live rabbits were caught in cage traps placed surrounding each warren as described in [34], with 50–60% of rabbits from each warren caught at each session [35]. Captured animals were handled at the trap-site, being marked with a numbered ear tag and having their sex and weight recorded. Females and males weighing more than 750 g and 850 g respectively were considered adults [36-38] (see Additional file 1 for details on sex and age population structures). Blood samples (1–2.5 mL) were collected from 1125 (17%) of 6605 captured animals over the course of the study. Details of the serology protocols employed are given in [34]. For both viruses, we considered seropositive those individuals with antibody concentrations sufficient to confer protection against disease as resulting from the ELISA test Information brochure (Ingezim Rabbit 1.7., Ingenasa Laboratory, Madrid, Spain). The probability of either false-positive or false-negative diagnoses under this paradigm are negligible (*P* < 0.02; [34]) making state misclassification unnecessary to be modeled. Furthermore, since antibodies against both viral diseases are detectable in blood for a short time (8–13 days) after exposure to either pathogen [39], seroprevalence to MV or RHDV provides a reliable insight into the epidemiological dynamics of the two diseases.

### Multi-event capture–recapture analyses

Capture–recapture sessions were not synchronized among the three fenced areas (E1–E3), for logistic reasons. As a result, we ran separate analyses for each enclosure and disease agent (MV and RHDV). With these populations serving as breeding zones for restocking purposes, random samples of captured individuals were periodically removed during capture sessions (removals of individuals is coded in the data sets and does not affect estimation of parameters). Sample sizes for capture–recapture analyses (i.e. number of captures minus number of removals) were 2573, 1771 and 1400 for E1–E3 respectively. Numbers of animals captured per trapping session, divided by the surface of the trapped enclosure, were used as indices of rabbit density [35] for each capture session and enclosure.

### Goodness of fit

Since no goodness of fit (GOF) is available for multi-event models, for each population we used U-CARE 2.3.2 [40] to test the fit of the Cormack–Jolly–Seber (CJS) model that accounted only for time variation in survival and capture probabilities. The CJS model is therefore more restrictive than those fitted for testing hypotheses that do account for the effect of serological status on recapture and transition probabilities. Hence, this approach is conservative given that if the CJS model adequately fits the data, then the multi-event models are also expected to fit. For each population/GOF analysis, we defined four groups according to age at first capture (adult vs. juvenile) and gender. U-CARE allows testing for specific lack of fit due to a transience effect (i.e. a higher than expected presence of individuals showing up only once; test component 3.SR) and/or to trap-dependence (i.e. capture probability depending on the fact they were captured or not; test component 2.CT). When the overall (all the groups and components together) goodness-of-fit tests were significant, sources of extra-binomial variation were accounted for in the multi-event global models by including transience and/or trap dependence (according to the output of tests 3.SR and 2.CT on each specific group). Over-dispersion factors (*c-hat*) were then calculated as the ratio between the sum of χ^2^ values and degrees of freedom of the non-significant test components [41].

For E1, the global goodness-of-fit test indicated lack of fit of the CJS model (χ^2^ = 112.47; d.f. = 87; *P* = 0.034), detecting “trap-happiness” among both adult males and females (*P* = 0.01 and < 0.01 respectively). For E2 and E3, there was no evidence of lack of fit (χ^2^ = 73.53; d.f. = 88; *P* = 0.87, and χ^2^ = 73.53; d.f. = 88; *P* = 0.8, respectively). We thus modeled trap-dependence among adults for E1 (see [42] and Additional file 2 for details on the probabilistic framework used). Correction for over-dispersion (*c-hat* = 1) was not needed for any analysis. Both survival and seroconversion rates have probably varied throughout the study period. However, because of limited sample sizes, and because our primary interest was in the net immunological effects on rabbit dynamics, we chose not to include a time effect on Survival and Seroconversion parameters.

### Multi-event design

During each field session (excluding the fifth, for logistical reasons) a variable and random sample of captured individuals were blood-sampled (regardless of sex, age, and encounter history) and their MV and RHDV immunological statuses (seropositive or seronegative) were assessed. To account for uncertainty in state-assessment when an individual is not bled (*sensu* “partial observation”; [27]) we used multi-event capture–recapture models [28] in E-SURGE 1.8.5 [43]. Unlike traditional methods for handling partial information on states, like data censoring or the extra-state approach [26], capture records in the multi-event framework are defined as events (i.e. reflecting the way the underlying biological states are observed in the field). It is therefore possible to define a specific event to record the capture of an individual whose state is unknown. Here we considered four events (not seen, 0; seen, bled and assessed as seronegative, 1; seen, bled and assessed as seropositive, 2; seen and not bled, 3) and three possible states (dead, †; alive seronegative, SN; alive seropositive, SP). A slightly different set of states was used in models accounting for trap-dependence (details on the probabilistic framework are given in Additional file 2).

Multi-event models include three parameter types, Initial State (related to the probability of being in some specific state when first captured), Transition (related to the probability of transition between states) and Event (related to the probabilities of being re-sighted according to the event-mediated information on states). We decomposed Transition into two steps: Survival (the survival probability) and, conditional on still being alive, Seroconversion (the seroconversion probability). Event was decomposed into two steps: Capture (accounting for recapture probability) and, conditional on being captured, State Assignment (accounting for the probability the immunological status was assessed). In this study, Initial State estimates the probability one first-captured individual is seropositive. Therefore, by assuming that the probability of first captured individuals being seropostive reflects the percentage of seropositive individuals in the population (but see [44,45] for a discussion on this), Initial State can be a proxy for the seroprevalence in each population.

We ran six analyses, one for each population–disease combination. We used QAICc values [46], to test for effects of immunity, age and sex on both rabbit survival and seroconversion rates (from seropositive to seronegative, and vice versa). Since populations were closed to immigration and emigration, survival rates referred to real survival rates [47]. We assumed that time intervals were short enough that multiple transitions between serological states were unlikely to occur between two consecutive sessions and no bias was expected on seroconversion estimates [48]. In E-SURGE we marked the “uneven time intervals” option to allow monthly estimates of both survival and seroconversion probabilities even though intervals between capture sessions were not on a monthly basis. We also used the best model from each analysis to test the effect of population density on Initial State (seroprevalence). For each analysis, we computed the significance and percentage of Initial State variation explained by density using analysis of deviance (ANODEV) [49]. This procedure compares the deviance and number of estimable parameters of three models identical except for the parameter of interest (Initial State in this case) which is: (*i*) constant, (*ii*) full-time dependent, or (*iii*) dependent on density.

### Model selection

Based on preliminary model exploration, Initial State and State Assignment depended on, respectively, time and time-by-immunological status and were not further modeled. The other parameters of the global model accounted for these effects: (*i*) Survival on age-by-sex-by-immunological status, (*ii*) Seroconversion on sex-by-immunological status; Capture on sex plus age-by-immunological status-by-time (in E1 also on trap-response).

For each population–disease combination, we first modeled recapture probabilities. The structure for recapture probabilities was then fixed as per the model with the lowest QAICc value, and Survival and Seroconversion probabilities were modeled independently. While we modeled Survival we kept the most parameterized structure for Seroconversion, and vice versa. For each parameter we considered a set of candidate models made of models nested to the global model. To keep the number of tested models as low as possible [46], we only considered interactive effects for parameters whose time-variation was not modeled (i.e. Survival and Seroconversion).

A final set of models combined the best structures for both Survival and Seroconversion (lowest QAICc when modeled independently) ([50], for a similar approach). Hence we used this set of models to compute, for each parameter, model-averaged estimates from models lying within 2 Δ of the best model [46].

## Results

### Myxoma virus and survival

**Figure 1:**
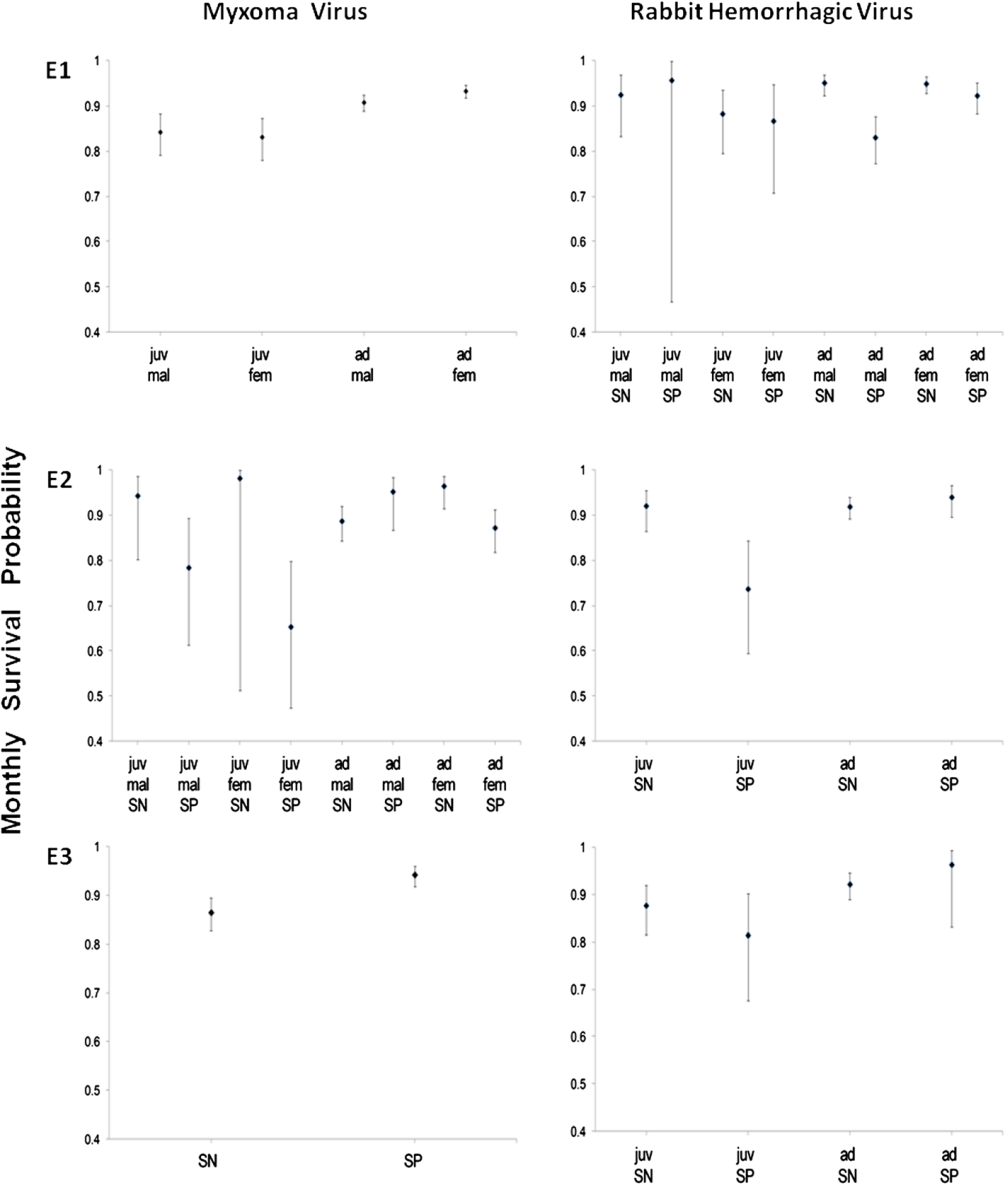
**Average monthly survival probability according to serological status (to MV and RHDV), age and gender in the three enclosures (E1, E2, and E3).** Only estimates related to effects selected in models with lowest QAICc are shown (e.g. if sex effect on survival was found to be negligible by model selection, one common estimate is shown for both males and females). Estimates with 95% confidence intervals are shown. Notation: juv, juvenile; SN, seronegative; SP, seropositive; mal, males; fem, females.

The relationship between MV seropositivity and survival varied among populations (Table 1, Figure 1, and Additional file 3 for numerical values of parameters). In E1, rabbit survival was variable among the age and sex classes but not between seropositives and seronegatives. In E2, MV-seropositive juveniles appeared to have lower survival rates than seronegatives (on average 25.1% lower, hereafter percentage differences refer to point estimates) but estimates were very imprecise; the same pattern was more evident among adult females (seropositive survival 9.6% lower) but the opposite trend was found among adult males (seropositive survival 6.8% higher). In E3, seropositives had higher survival rates than seronegatives in all age and sex classes (8.2% higher).

**Table 1:** Myxoma Virus model-selection best models

**Enclosure**	**Parameter**	**Model effects**	**np; Dev; QAIC**_**c**_**; w**_**i**_
**E1**	Survival	Age*sex	58; 6694.55; 6813.05; 0.66
		Age	56; 6700.78; 6815.10; 0.23
		Age*immun	58; 6699.49; 6817.99; 0.06
	Seroconversion	Sex*immun	62; 6691.23; 6818.08; 0.92
**E2**	Survival	Age*sex*immun	53; 4733.87; 4053.88; 0.88
		Age*immun	49; 4750.48; 4059.29; 0.06
	Serocoversion	Sex*immun	53; 4733.87; 4053.88; 1
**E3**	Survival	Immun	48; 3664.89; 3764.20; 0.69
		Age*sex*immun	54; 3654.65; 3766.85; 0.18
		Sex*immun	50; 3664.83; 3768.43; 0.08
	Seroconversion	Immun	52; 3656.86; 3764.76; 0.71
		Sex*immun	54; 3654.65; 3766.85; 0.25

np, number of parameters; Dev, Deviance; QAIC_c_, Quasi-Akaike Information Criterion corrected for over-dispersion; w_*i*_, Akaike weight (support of the current model with respect to the candidate set of models). Model notation: immun, the immunological status: for survival it means a different survival rate according to immunological status whereas for seroconversion it means that seroconversion rate from seropositive to seronegative is different than seroconversion from seronegative to seropositive; *age*, juveniles *vs*. adults; *sex*, females *vs*. males.

For each analysis, only models accounting for more than 90% of cumulative Akaike weights are reported.

### Rabbit hemorrhagic disease and survival

The relationship between RHDV seropositivity and survival also varied among populations (Table 2, Figure 1, and Additional file 3). However, there was a general pattern of seropositives tending to have lower survival than seronegatives. In E1, there was no clear relationship between RHDV seropositivity and either juvenile or adult female survival, whereas it was related to lower survival (12.6%) in adult males. In E2, seropositivity was related to lower juvenile survival (20%), but no relationship was observed for adults. In E3, seropositive juveniles appeared to survive less (7.1%) than seronegatives, whereas no clear effect was found for adults.

**Table 2:** Rabbit Hemorrhagic Disease Virus model-selection best models

**Enclosure**	**Parameter**	**Model effects**	**np; Dev; QAIC**_**c**_**; w**_**i**_
**E1**	Survival	Age*sex*immun	83; 6518.66; 6689.61; 0.73
		Sex*immun	79; 6530.35; 6692.83; 0.15
		Immun	77; 6535.99; 6694.24; 0.07
	Seroconversion	Immun	81; 6519.89; 6686.59; 0.74
		Sex*immun	83; 6518.66; 6689.61; 0.16
**E2**	Survival	Age*immun	49; 4724.47; 4037.61; 0.62
		Age*sex*immun	53; 4718.07; 4040.71; 0.13
		Age	47; 4733.35; 4040.81; 0.13
		Age*sex	49; 4729.07; 4041.44; 0.09
	Serocoversion	Sex*immun	53; 4718.07; 4040.71; 0.82
		Sex	51; 4726.87; 4043.82; 0.17
**E3**	Survival	Age*immun	42; 3830.64; 3917.17; 0.42
		Age	40; 3835.32; 3917.62; 0.34
		Immun	40; 3837.85; 3920.15; 0.09
		Age*sex	42; 3834.10; 3920.63; 0.07
	Seroconversion	Sex*immun	46; 3827.61; 3922.65; 0.54
		Immun	44; 3832.35; 3923.13; 0.43

See Table 1 footnote for notations used.

### Myxoma virus seroconversion

**Figure 2:**
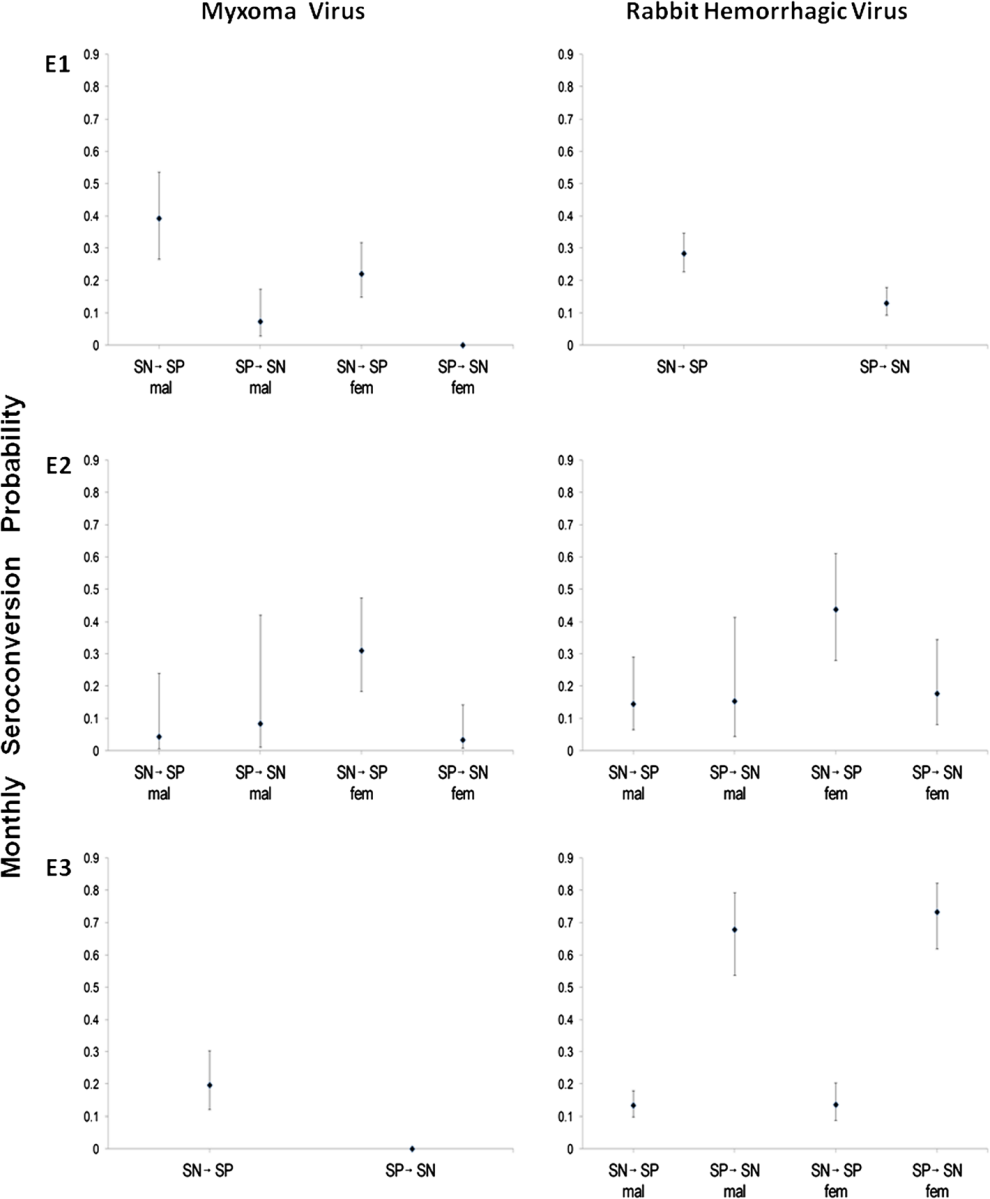
**Myxoma and Rabbit Hemorrhagic Disease Virus seroconversion rates in the three enclosures (E1, E2, and E3).** Estimates with 95% confidence intervals are shown. Notation: SN → SP, average monthly rate at which individuals change their immunological status from seronegative to seropositive; SP → SN, from seropositive to seronegative; mal, males; fem, females.

Overall, the probability of becoming MV seropositive was higher than that of becoming seronegative (Table 1, Figure 2, and Additional file 4 for numerical values of parameters). In E1, both males and females became seropositive at a faster rate than the reverse; for females the probability of becoming seronegative was null suggesting that female rabbits once seropositive remain so. Males appeared to become seropositive at a faster rate than females. In E2, females became seropositive at a faster rate than the reverse, and faster than males. In E3, the probability of becoming seropositive was the same for both genders, and the reverse was null.

### Rabbit Hemorrhagic Disease Virus seroconversion

We found very different patterns of RHDV seroconversion among enclosures (Table 2, Figure 2, and Additional file 4). In E1, no difference was observed among genders, with all rabbits becoming seropositive at a faster rate than the reverse. In E2, there were no consistent differences in seroconversion rates of males, while females became seropositive at a higher rate than the reverse (and faster than males). In contrast, in E3 both sexes became seropositive at an unusually high rate, and faster than they became seronegative (from model without sex effect: respectively, 0.72, SE: 0.05; 0.14, SE: 0.02).

### Seroprevalence and density

Seroprevalence to myxoma and RHD viruses varied across time in all populations (Table 3, and Additional files 5, 6 and 7, respectively), but high levels of uncertainty on several estimates made it difficult to discern time-trends.

**Table 3:** Effect of rabbit density on Initial State (seroprevalence of MV and RHDV)

	**Myxo – Prev**	**RHD – Prev**
**E1**	4.65; 0.07; 0.66	5.11; 0.06; 0.73
**E2**	0.25; 0.63; 0.03	0.80; 0.4; 0.11
**E3**	5.01; 0.06; 0.71	2.94; 0.13; 0.42

From left to right: the Fisher–Snedecor statistic (F_1,7_ for Initial State and F_1,6_ for the others), *P*-value (in bold if < 0.1), and R^2^. All statistics were computed following the ANODEV procedure. Myxo, myxoma virus; RHD, Rabbit Hemorrhagic Disease Virus; Prev, prevalence.

Population size in the three enclosures followed similar patterns, being lower during the winter and higher during the spring. The three populations reached maximum peak densities at the same time in May 2010, while minimum densities occurred at different times. Average rabbit densities (no./hectare) per enclosure (± 95% CI) over the whole study period were 79.6 ± 53.1, 49.9 ± 31.4, and 52.5 ± 38.5 (for E1–E3 respectively). We observed a marginally significant positive relationship between rabbit density and MV seroprevalence for E1 (slope on logit scale: 1.73; SE: 0.3; *P*: 0.07; 66% variation explained), and a negative such relationship for E3 (slope on logit scale: -1.13; SE: 0.26; *P*: 0.06; 71% variation explained). We also observed a marginally significant positive relationship between density and RHDV seroprevalence in E1 (slope on logit scale: 1.41; SE: 0.17; *P*: 0.06; 73% variation explained).

### Recapture probability

Recapture rates varied through time in all analyses (average estimates ± SD from best models of MV analyses: E1, 0.61 ± 0.22; E2, 0.48 ± 0.19; E3, 0.28 ± 0.19). We found either sex, age, or immunological status affected the probability of being recaptured (in E1 trap happiness was also confirmed throughout model selection). However, very heterogeneous causal effects on recapture probabilities were found among and within the rabbit populations.

## Discussion

Our study confirms that rabbit populations respond very differently in terms of host survival and epidemiological dynamics when exposed to MV and RHDV, even if geographically close to each other [51,52]. Multiple interacting causes likely explain this heterogeneity. However, some general patterns were observed in our study: (*i*) RHDV seropositivity was never related to increased rabbit survival; (*ii*) seropositivity to both MV and RHDV was negatively related to survival more frequently for juveniles than for adults; and (*iii*) once MV seropositive, rabbits rarely lost such status.

### Survival

Our results do not conform to the overall published pattern of MV and RHDV seropositive rabbits always having higher survival rates than seronegatives [29-31]. Only in one case, MV seropositives had considerably higher survival rates than seronegatives. However, it should be noted that our populations were in semi-natural captive conditions allowing high densities, which greatly exceeded wild population densities (e.g. *c*. 3 rabbits/ha for the central Iberian Peninsula; [53]). Therefore our results could be extrapolated to high density scenarios in the wild (e.g. [54,55]).

Seropositivity to MV and RHDV could potentially confer both advantages and disadvantages to rabbits. While it may confer higher immunity to these pathogens, several authors have recognized that (for MV at least) seropositivity has an immunosuppressive effect that may cause higher rates of co-infection with other pathogens [56-58]. With the target organ of RHDV (the liver) being part of the immune system, RHDV may also cause an immunosuppressive syndrome in addition to reduced survival due to hemorrhagic effects [59-61]. Rabbits seropositive to either virus are thus likely to be more susceptible to damage from other infections. A shifting balance of advantages and disadvantages is thus one explanation for contrasting patterns, and is likely influenced by interactions with other environmental, epidemiologic and individual factors. For example, in our population E3 rabbits were in a better physiological status than in the others as a result of availability of higher water quality (e.g. in streams, manuscript in preparation). We suggest that this reduced the negative effect of the MV-seropositive-related immunosuppression, explaining why they had higher survival rates than seronegatives. As another example, RHDV seropositivity never being associated with higher survival rates could indicate that the negative effects of the hemorrhagic syndrome outweighed any beneficial effects. Finally, seropositive juveniles tending to survive less than seronegatives may be explained by the immaturity of the juvenile immune system with full immunity not yet being fully acquired. In light of this, it is possible that negative influences such as immunosuppression and the hemorrhagic syndrome of RHDV had greater influence on juveniles than adults.

### Seroconversion

With ongoing persistence 50 and 30 years after the arrival of myxomatosis and RHD respectively on the Iberian Peninsula [62], it is reasonable to classify the viruses as endemic. Accordingly, rabbits should gain MV and RHD antibodies as fast as or faster than they lose them. Seroconversion to seropositive status tending to occur at a faster rate than the reverse in our study (and also [62] and [31]) supports this expectation. However, this was not the case for RHDV in E3, where seroconversion to seronegative status occurred at a higher rate. We suggest this result does not disprove the endemic disease behavior hypothesized above, but is driven by asynchrony between population and virus dynamics. In fact, RHDV was found in this population over the 7 years of monitoring (SM, unpublished data), but the population had a delayed breeding season that, differently from in the other enclosures, occurred after the RHD outbreak. Thus, in E3 the naïve kittens would have lost their maternal antibodies after 2 months [63] and, since they were not exposed to RHDV, a large number of seronegative adults (many of them born the spring just before) would have occurred in the next autumn. This would result in a large number of seronegatives over the study period and explain this seemingly inconsistent result. It should also be noted that, in contrast to general belief [64], the average monthly probability of losing antibodies was not necessarily null for either RHDV or MV. This indicates that rabbits can lose immunity to these diseases (albeit with a very low probability). The effect of sex on the rate at which individuals became seropositive varying among populations further illustrates the varying behavior of these diseases across individuals and populations [51,52].

### Antibody prevalence and density

In general, seroprevalence did not follow a consistent pattern either within or among populations for either disease. This was in agreement with previous findings from some authors, stating these diseases behave very differently among populations [51,52], but contrasts with a study in the Canary Islands [65] where they found no difference in RHDV prevalence across four neighboring geographic zones.

Host infection by both viruses also occurs by means of contact with an infected individual. Population size is thus recognized as an important factor promoting MV and RHDV dynamics [52,66-68]. However, even though in some cases a great amount of variation in seroprevalence appeared to be explained by density, in no case did we find this hypothesis was strongly supported (*P*-values were marginally significant at the 0.05 level). The pattern of increasing prevalence with population size observed in E1 for both MV and RHDV was unsurprising. However, we observed no such relationships in E2 and a negative relationship between MV prevalence and population size in E3. This last result may have been caused by a correlation between decreasing density due to gradual habitat degradation (authors personal observation) leading to individuals under nutritional stress being more susceptible to infection.

### Conclusion

This is the first multi-event study focusing on MV and RHDV host–pathogen dynamics in rabbit populations. We found that while MV seropositivity had either a positive or negative effect on survival that was likely dependent on interaction with other factors (e.g. physiological condition), the hemorrhagic syndrome caused by RHDV led seropositive rabbits to suffer higher mortality rates. Our study highlights that the host–pathogen dynamics of these viruses are highly variable among populations even when these share similar management and climatic conditions. These findings have important implications for rabbit population management, particularly where their scarcity could compromise ecosystem conservation. Additional well-defined capture–recapture analyses may shed further light on the still many obscure mechanisms driving host–pathogen dynamics (e.g. [69,70]).

## Competing interests

The authors declare that they have no competing interests.

## Authors’ contributions

CR and SS conceived and designed the study. IP and SM participated in the design of the study. CR, IP, SM and AB assisted in the field with animal handling and collected and processed the samples. AB conducted the serological analysis. SS carried out all statistical analysis. CR, SS and IP drafted the manuscript and SM and AB helped edit the manuscript. All authors read and approved the final manuscript.

## Supplementary Material

Additional file 1 **Sex and age population structures in each enclosure (E1, E2 and E3).** Frequency of males and females, juveniles and adults, as resulting from captured individuals for each session capture in the three enclosures. Click here for file

Additional file 2 **Probabilistic framework of the multi-event analyses.** Matrice structure for each parameter (Initial State, Survival, Seroconversion, Capture or Trap-Dependence, and State Assignment) is given and its notation explained. Click here for file

Additional file 3 **Survival estimates with 95% CI for each enclosure (E1, E2 and E3).** Survival estimates in the three enclosures as depending on the main effects found through model selection. Click here for file

Additional file 4 **Seroconversion estimates with 95% CI for each enclosure (E1, E2 and E3).** Seroconversion estimates in the three enclosures as depending on the main effects found through model selection. No age effect was tested because juveniles remain juveniles only for a short time and no recapture from one individual in juvenile state exists. Click here for file

Additional file 5 **Myxoma virus and RHDV antibody prevalence in enclosure E1.** Myxoma virus and Rabbit Hemorrhagic Disease Virus seroprevalences over the study period as proxied by Initial State in enclosure E1. Prevalence estimates refer to the probability one first-captured individual is seropositive. This probability is corrected for the specific session probability of capture of seronegatives and seropositives. Vertical bars represents 95% confidence intervals. Click here for file

Additional file 6 **Myxoma virus and RHDV antibody prevalence in enclosure E2.** Myxoma virus and Rabbit Hemorrhagic Disease Virus seroprevalences over the study period as proxied by Initial State in enclosure E2. Prevalence estimates refer to the probability one first-captured individual is seropositive. This probability is corrected for the specific session probability of capture of seronegatives and seropositives. Vertical bars represent 95% confidence intervals. Click here for file

Additional file 7 **Myxoma virus and RHDV antibody prevalence in enclosure E3.** Myxoma virus and Rabbit Hemorrhagic Disease Virus seroprevalences over the study period as proxied by Initial State in enclosure E3. Prevalence estimates refer to the probability one first-captured individual is seropositive. This probability is corrected for the specific session probability of capture of seronegatives and seropositives. Vertical bars represent 95% confidence intervals. Click here for file
