# Supplementary figures and images for: Multi-event capture–recapture modeling of host–pathogen dynamics among European rabbit populations exposed to myxoma and Rabbit Hemorrhagic Disease Viruses: common and heterogeneous patterns
Source: Vet Res. 2014 Apr 5;45(1):39. doi: 10.1186/1297-9716-45-39 (PMC4021418; doi:10.1186/1297-9716-45-39)

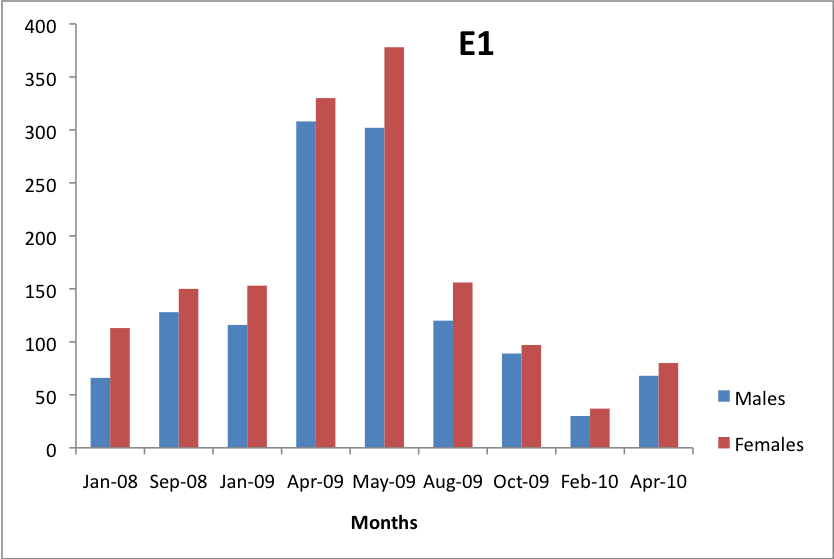


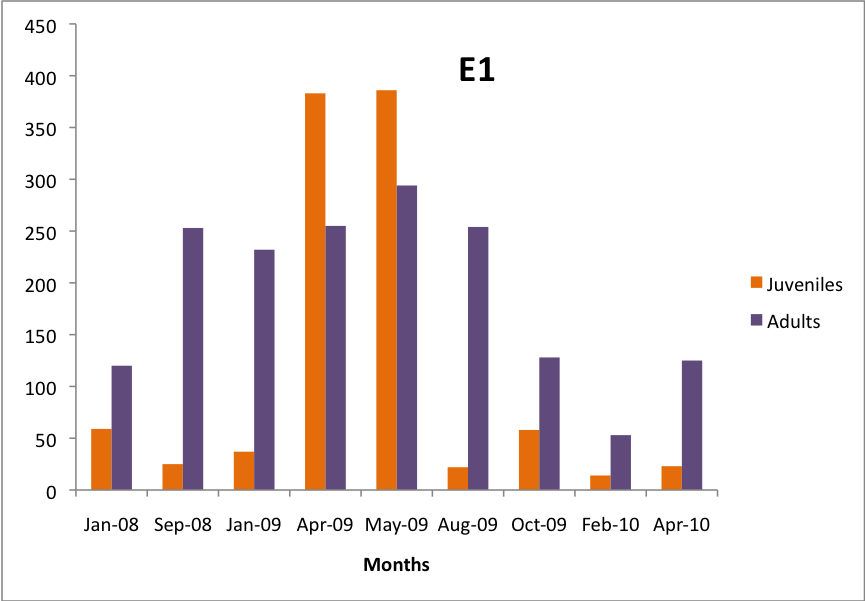


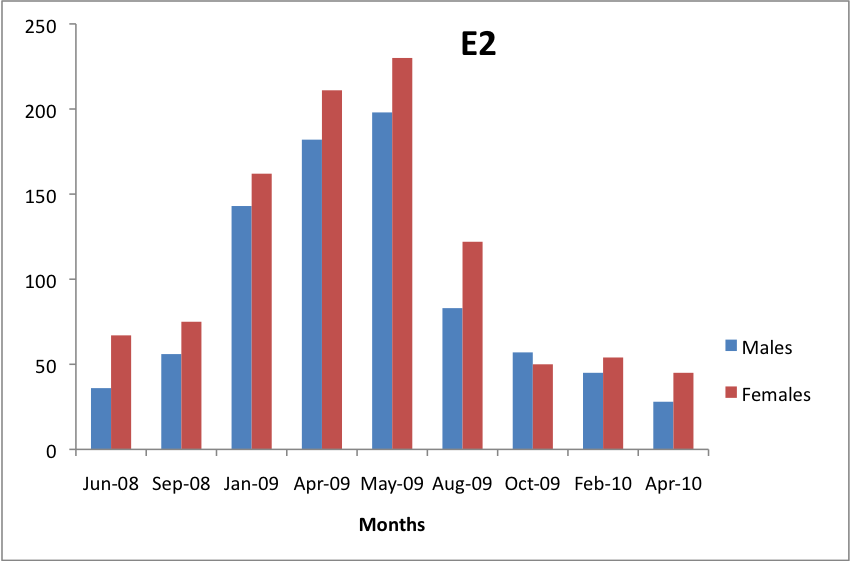


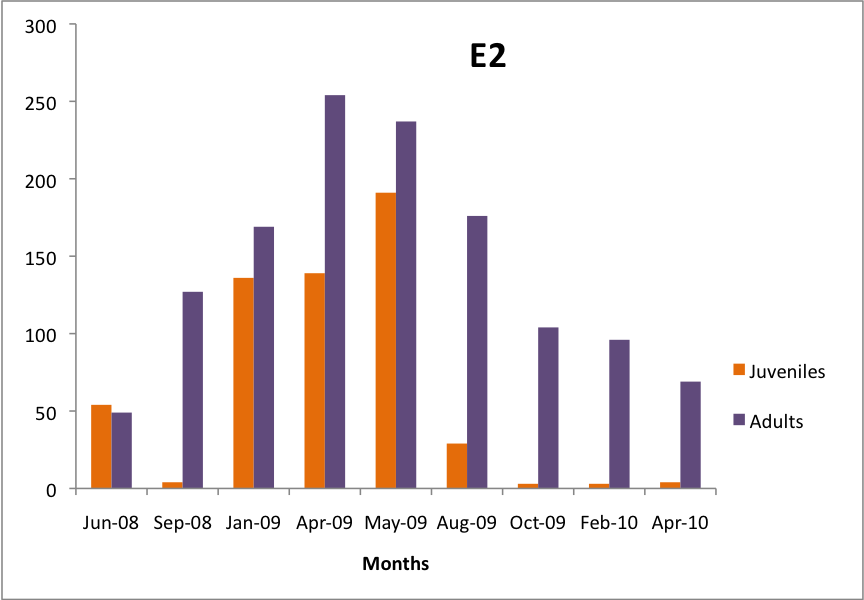


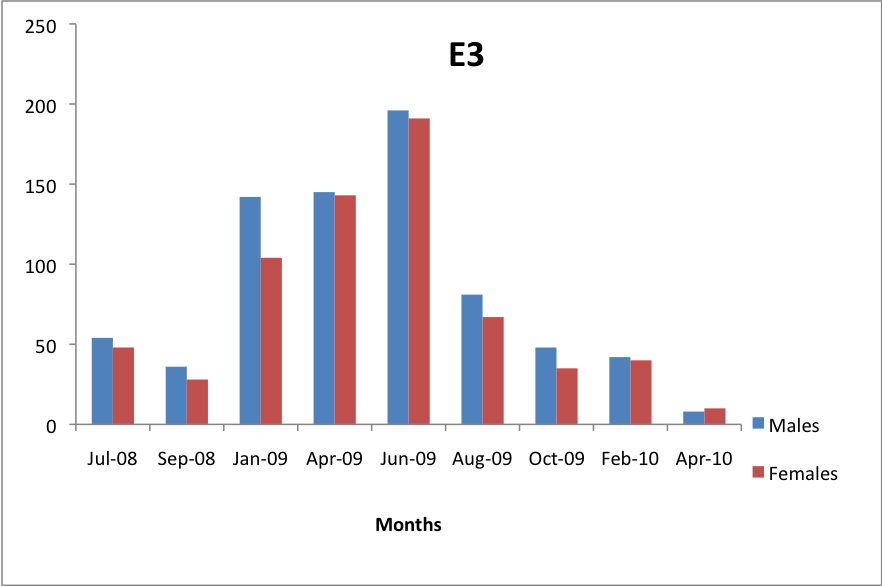


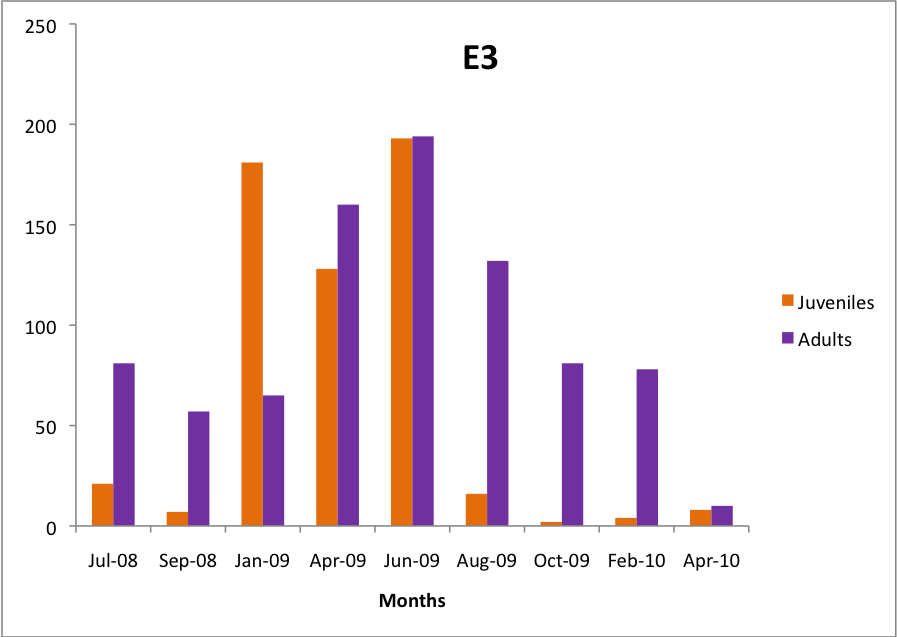

Supplement: Additional file 1 — Sex and age population structures in each enclosure (E1, E2 and E3). Frequency of males and females, juveniles and adults, as resulting from captured individuals for each session capture in the three enclosures. [file 1297-9716-45-39-S1.doc]

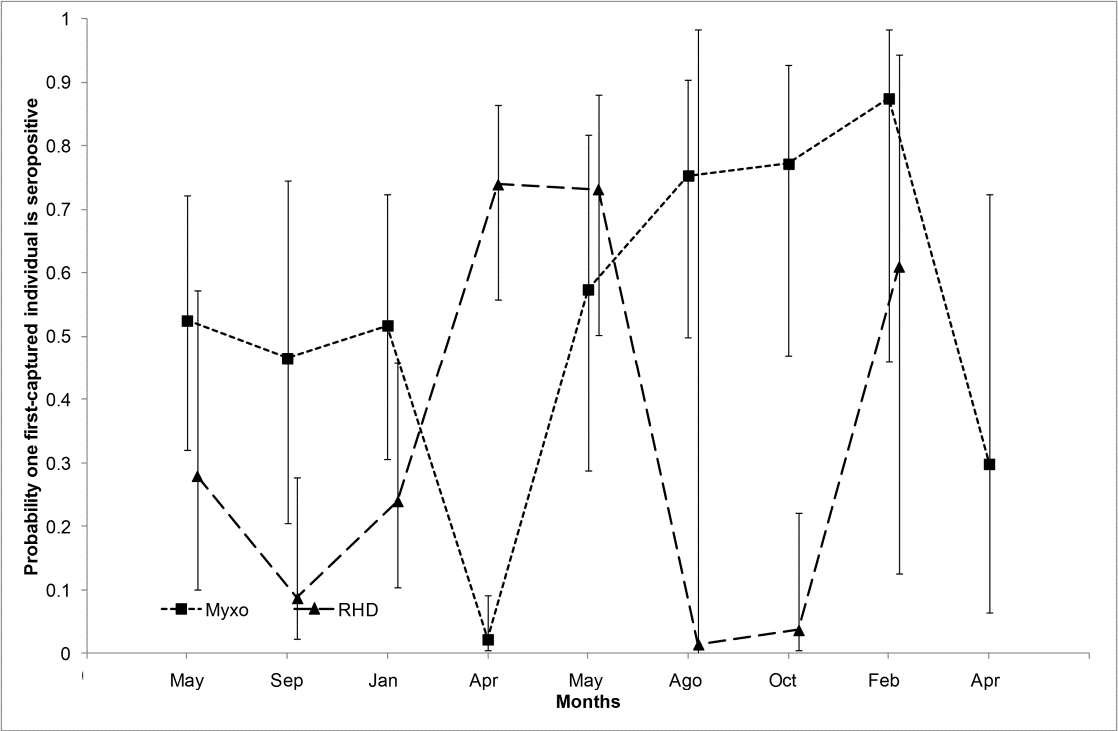

Supplement: Additional file 5 — Myxoma virus and RHDV antibody prevalence in enclosure E1. Myxoma virus and Rabbit Hemorrhagic Disease Virus seroprevalences over the study period as proxied by Initial State in enclosure E1. Prevalence estimates refer to the probability one first-captured individual is seropositive. This probability is corrected for the specific session probability of capture of seronegatives and seropositives. Vertical bars represents 95% confidence intervals. [file 1297-9716-45-39-S5.doc]

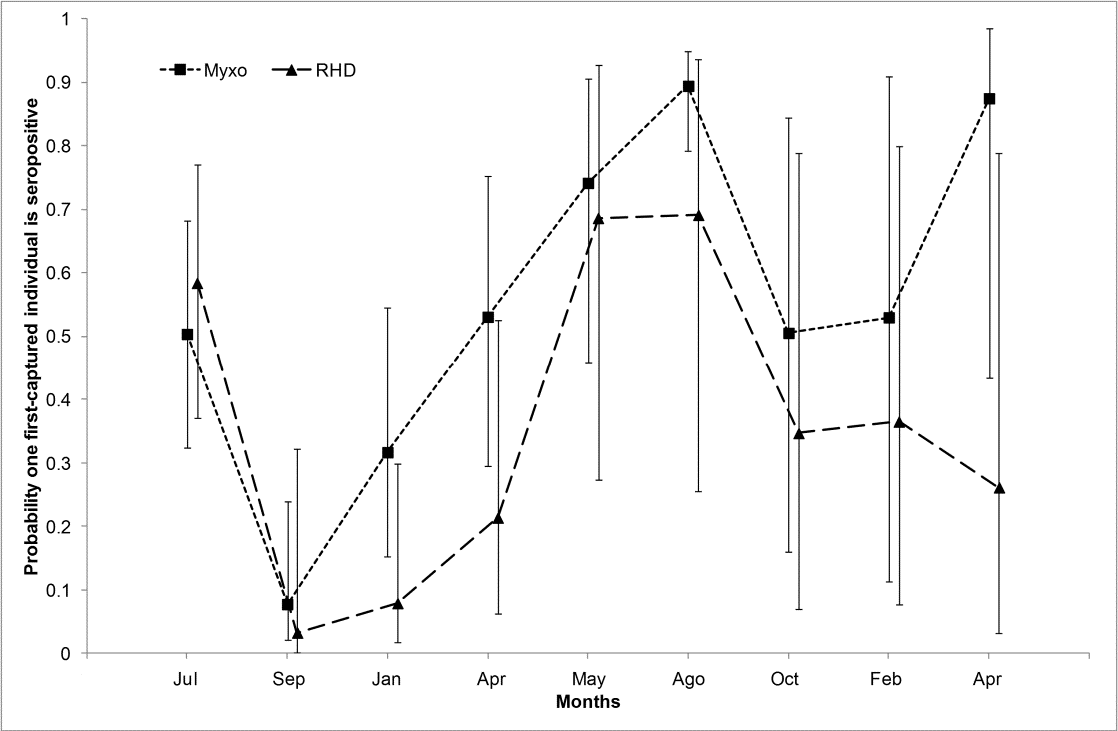

Supplement: Additional file 6 — Myxoma virus and RHDV antibody prevalence in enclosure E2. Myxoma virus and Rabbit Hemorrhagic Disease Virus seroprevalences over the study period as proxied by Initial State in enclosure E2. Prevalence estimates refer to the probability one first-captured individual is seropositive. This probability is corrected for the specific session probability of capture of seronegatives and seropositives. Vertical bars represent 95% confidence intervals. [file 1297-9716-45-39-S6.doc]

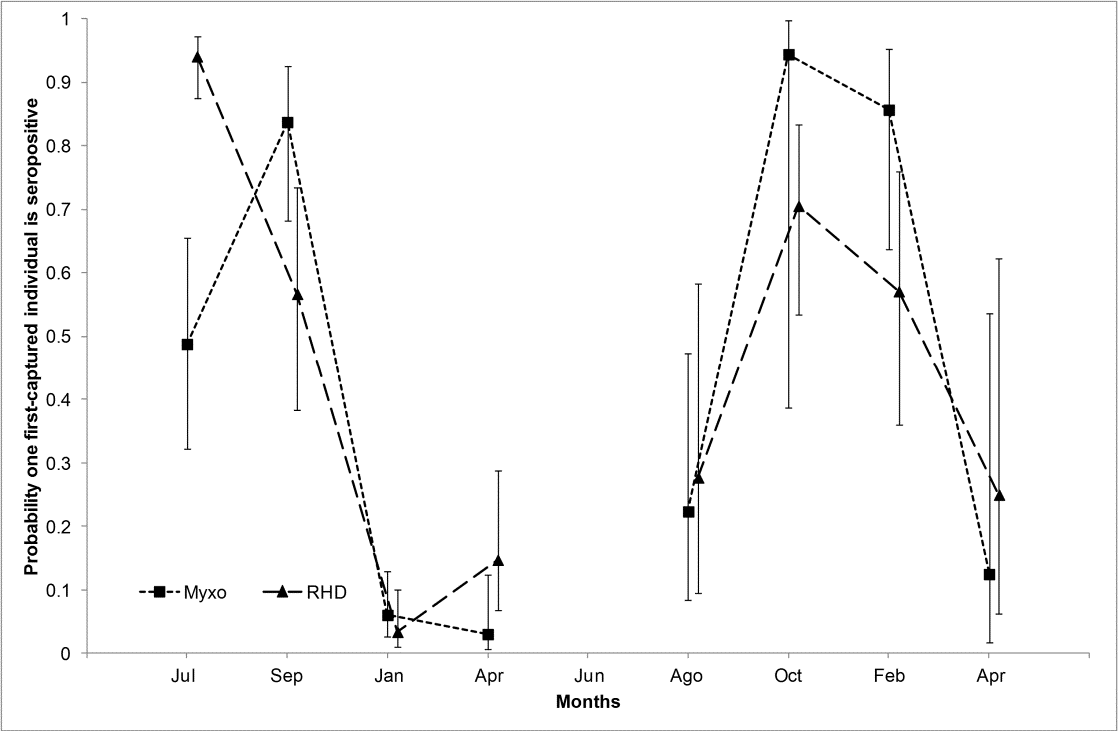

Supplement: Additional file 7 — Myxoma virus and RHDV antibody prevalence in enclosure E3. Myxoma virus and Rabbit Hemorrhagic Disease Virus seroprevalences over the study period as proxied by Initial State in enclosure E3. Prevalence estimates refer to the probability one first-captured individual is seropositive. This probability is corrected for the specific session probability of capture of seronegatives and seropositives. Vertical bars represent 95% confidence intervals. [file 1297-9716-45-39-S7.doc]
