# Supplementary material for: Multi-event capture–recapture modeling of host–pathogen dynamics among European rabbit populations exposed to myxoma and Rabbit Hemorrhagic Disease Viruses: common and heterogeneous patterns
Source: Vet Res. 2014 Apr 5;45(1):39. doi: 10.1186/1297-9716-45-39 (PMC4021418; doi:10.1186/1297-9716-45-39)
Supplement: Additional file 2 — Probabilistic framework of the multi-event analyses. Matrice structure for each parameter (Initial State, Survival, Seroconversion, Capture or Trap-Dependence, and State Assignment) is given and its notation explained. [file 1297-9716-45-39-S2.doc]

**Multievent probabilistic framework of the study**

Multievent models combine information from events with the underlying states to estimate probabilities of several parameters. A multievent model accounts for three parameter types: Initial State, State Transition and Event probabilities.

In this study we defined three underlying biological states:

✝ - Death

SN - Seronegative alive

SP - Seropositive alive

And four events, numbered as they appear in the data set:

0 - Rabbit not captured

1 - Rabbit captured, bled and classified as seronegative

2 - Rabbit captured, bled and classified as seropositive

3 - Rabbit captured, not bled

**Groups:**

Groups are coded within the “headed format” data file (see [43]) as: (1) Juvenile Male, (2) Juvenile Female, (3) Adult Male, and (4) Adult Female.

**INITIAL STATE**

This parameter refers to the probability that, an individual is seropositive when first captured. As stated by Pradel [28]: “*The “initial state probabilities”, [...] describe the probability that an individual is in one or another state when it is first encountered [...] This is an entirely new type of parameter from multistate models. Dependent on the kind of study, it can be related to the sex-ratio, the prevalence of a disease or the percentage of breeders in the population*”.

This parameter here relates to the proportion of seropositives among the new unmarked individuals captured. By assuming that (*i*) the serological status does not seriously affect the probability of being captured, and (*ii*) the sampling scheme has not varied during the study period, this parameter can be related to the seroprevalence of the population (see [28,45] for a discussion on this). By definition, the probability of being first captured as a death individual is zero. Therefore, the initial state probabilities are:

| SN | SP |
| --- | --- |
| 1-π | π |

where π is the probability, at a certain occasion *t*, a first captured rabbit has to be seropositive (note that no age or sex effect has been modeled on this parameter).

**STATE TRANSITION**

This parameter type was divided in two steps. These probabilities are best represented in the form of stochastic matrices with departure states in rows and arrival states in columns.

**Step 1 Survival**; this step computes the probability that an individual first captured on occasion *t* survives in the interval between *t* and *t* + 1.

|  | SN | SP | ✝ |
| --- | --- | --- | --- |
| SN | φ_SN_ | 0 | 1-φ_SN_ |
| SP | 0 | φ_SP_ | 1-φ_SP_ |
| ✝ | 0 | 0 | 1 |

Where φ_SN_ and φ_SP_ refer respectively to the probability a seronegative or a seropositive has of surviving in the interval after its first capture.

**Step 2 Seroconversion (conditional on Survival)**; this step computes the probability a survived individual has of changing its state between *t* and *t* + 1.

|  | SN | SP | ✝ |
| --- | --- | --- | --- |
| SN | 1-ψ_SN_ | ψ_SN_ | 0 |
| SP | ψ_SP_ | 1-ψ_SP_ | 0 |
| ✝ | 0 | 0 | 1 |

Where, conditioned on having survived the current interval, ψ_SN_ and ψ_SP_ refer respectively to the probability a seronegative has to become a seropositive and a seropositive has to become a seronegative.

**EVENT**

The Event probabilities relate the events to the underlying biological states. This parameter type has been divided into two steps.

**Step 1 Capture.** This estimates the probability one individual has to be recaptured at *t* + 1.

|  | not captured | SN captured | SP captured |
| --- | --- | --- | --- |
| SN | 1 - β_SN_ | β_SN_ | 0 |
| SP | 1 - β_SP_ | 0 | β_SP_ |
| ✝ | 1 | 0 | 0 |

Where β_SN_ and β_SP_ refer respectively to the probability a seronegative and a seropositive have to be captured.

**Step 2 State Assignment (conditional on Capture)**; this estimates, conditional on being captured, the probability one individual has to be bled when captured. In columns are the events as they are coded in the data set.

|  | 0-not captured | 1-captured SN bled | 2-captured SP bled | 3-captured not bled |
| --- | --- | --- | --- | --- |
| not captured | 1 | 0 | 0 | 0 |
| SN captured | 0 | γ_SN_ | 0 | 1-γ_SN_ |
| SP captured | 0 | 0 | γ_SP_ | 1 - γ_SP_ |

Where, conditioned on have being captured the current session, γ_SN_ and γ_SP_ refer respectively to the probability a seronegative and a seropositive have to be bled.

**TRAP-DEPENDENCE PARAMETERIZATION** (used for E1 data set)

When modeling trap-dependence (trap-happiness in our case) we followed the parameterization indicated by [42]. This parameterization consists of a modification of the number of states and an additional transition parameter step that specifically addresses trap-dependence. States are hence defined according to the fact one individual was captured just before (trap Aware) of the current session or not (trap Unaware). This way the capture probabilities appear as a step of transition parameters, more details are given in [42]. In our case we defined five underlying biological states:

✝ - Death

SN_(U)_ - Seronegative alive not captured at the previous session

SN_(A)_ - Seronegative alive captured at the previous session

SP_(U)_ - Seropositive alive not captured at the previous session

SP_(A)_ - Seropositive alive captured at the previous session

The events remained the same as before:

0 - Rabbit not captured

1 - Rabbit captured, bled and classified as seronegative

2 - Rabbit captured, bled and classified as seropositive

3 - Rabbit captured, not bled

INITIAL STATE

| SN_(U)_ | SN_(A)_ | SP_(U)_ | SP_(A)_ |
| --- | --- | --- | --- |
| 1-π | 0 | π | 0 |

where π is the probability, at a certain occasion *t*, a first captured rabbit has to be seropositive. Note that: (*i*) a previously captured individual has, by definition, zero probability of being first-time captured at the current occasion and, (*ii*) no age or sex effect has been modeled on this parameter.

**STATE TRANSITION**

**Step 1 Survival**; this step computes the probability that an individual first captured on occasion *t* survives in the interval between *t* and *t* + 1^-^ (the instant just before *t* + 1).

|  | SN_(U)_ | SN_(A)_ | SP_(U)_ | SP_(A)_ | ✝ |
| --- | --- | --- | --- | --- | --- |
| SN_(U)_ | φ_SN(U)_ | 0 | 0 | 0 | 1- φ_SN(U)_ |
| SN_(A)_ | 0 | φ_SN(A)_ | 0 | 0 | 1- φ_SN(A)_ |
| SP_(U)_ | 0 | 0 | φ_SP(U)_ | 0 | 1- φ_SP(U)_ |
| SP_(A)_ | 0 | 0 | 0 | φ_SP(A)_ | 1- φ_SP(A)_ |
| ✝ | 0 | 0 | 0 | 0 | 1 |

Where φ_SN_ and φ_SP_ refer respectively to the probability a seronegative or a seropositive has of surviving in the interval after its first capture and notation (U) and (A) refers respectively to individuals not captured (trap-unaware) and captured (trap-aware) at the previous occasion. In E-SURGE, we constrained φ_SN(U)_ = φ_SN(A)_ and φ_SP(U)_ = φ_SP(A)_ so that the trap-awareness process had no effect on survival probabilities.

**Step 2 Seroconversion (conditional on Survival)**; this step computes the probability a survived individual has of changing its state at *t* + 1^-^ (the instant just before t + 1).

|  | SN_(U)_ | SN_(A)_ | SP_(U)_ | SP_(A)_ | ✝ |
| --- | --- | --- | --- | --- | --- |
| SN_(U)_ | 1- ψ_SN(U)_ | 0 | ψ_SN(U)_ | 0 | 0 |
| SN_(A)_ | 0 | 1- ψ_SN(A)_ | 0 | ψ_SN(A)_ | 0 |
| SP_(U)_ | ψ_SP(U)_ | 0 | 1-ψ_SP(U)_ | 0 | 0 |
| SP_(A)_ | 0 | ψ_SP(A)_ | 0 | 1- ψ_SP(A)_ | 0 |
| ✝ | 0 | 0 | 0 | 0 | 1 |

Where, conditioned on having survived the current interval: (*i*) ψ_SN_ and ψ_SP_ refer respectively to the probability a seronegative has to become a seropositive and a seropositive has to become a seronegative and, (*ii*) notation “(U)” and “(A)” have the same meaning as from step 1. In E-SURGE, we have constrained ψ_SN(U)_ = ψ_SN(A)_ and ψ_SP(U)_ = ψ_SP(A)_.

**Step 3 Trap-Dependence (conditional on Survival and Seroconversion)**; this step computes the probability an individual alive and in a certain serological state at *t* + 1^-^ (the instant just before *t* + 1) has of changing its trap-awareness state between *t* + 1^-^ and *t* + 1^+^, i.e. between the instant just before the *t* + 1 and the instant just after it.

|  | SN_(U)_ | SN_(A)_ | SP_(U)_ | SP_(A)_ | ✝ |
| --- | --- | --- | --- | --- | --- |
| SN_(U)_ | 1-τ_SN(U)_ | τ_SN(U)_ | 0 | 0 | 0 |
| SN_(A)_ | 1-τ_SN(A)_ | τ_SN(A)_ | 0 | 0 | 0 |
| SP_(U)_ | 0 | 0 | 1-τ_SP(U)_ | τ_SP(U)_ | 0 |
| SP_(A)_ | 0 | 0 | 1-τ_SP(A)_ | τ_SP(A)_ | 0 |
| ✝ | 0 | 0 | 0 | 0 | 1 |

Where, conditioned on having survived the current interval and being in a certain serological state in *t* + 1^-^ : (*i*) τ_SN_ and τ_SP_ refer respectively to the probability of being trap-aware at *t* +1^+^ , i.e. of being captured at *t* + 1, and, (*ii*) notation "(U)" and "(A)" have the same meaning as from previous step 1.

**EVENT**

The Event probabilities here relates uniquely to the State Assignment process.

**Step 1 State Assignment (conditional on Capture)**; this step computes, conditional on being captured at *t* + 1 (see step 3 in Transition), the probability one individual has to be bled when captured. In columns are the events as they are coded in the data set.

|  | 0-not captured | 1-captured SN bled | 2-captured SP bled | 3-captured not bled |
| --- | --- | --- | --- | --- |
| SN_(U)_ | 1 | 0 | 0 | 0 |
| SN_(A)_ | 0 | γ_SN_ | 0 | 1-γ_SN_ |
| SP_(U)_ | 1 | 0 | 0 | 0 |
| SP_(A)_ | 0 | 0 | γ_SP_ | 1 - γ_SP_ |
| ✝ | 1 | 0 | 0 | 0 |

Where, conditioned on have being captured the current session, γ_SN_ and γ_SP_ refer respectively to the probability a seronegative and a seropositive have to be bled.
