# Supplementary material for: Multi-event capture–recapture modeling of host–pathogen dynamics among European rabbit populations exposed to myxoma and Rabbit Hemorrhagic Disease Viruses: common and heterogeneous patterns
Source: Vet Res. 2014 Apr 5;45(1):39. doi: 10.1186/1297-9716-45-39 (PMC4021418; doi:10.1186/1297-9716-45-39)
Supplement: Additional file 3 — Survival estimates with 95% CI for each enclosure (E1, E2 and E3). Survival estimates in the three enclosures as depending on the main effects found through model selection. [file 1297-9716-45-39-S3.doc]

| **Main effects** | **Estimates** | **Low 95% CI** | **High 95% CI** |
| --- | --- | --- | --- |
| **E1 MYXO** |  |  |  |
| juv mal | 0.84 | 0.79 | 0.88 |
| juv fem | 0.83 | 0.78 | 0.87 |
| ad mal | 0.91 | 0.89 | 0.92 |
| ad fem | 0.93 | 0.92 | 0.95 |
| **E1 RHD** |  |  |  |
| juv mal SN | 0.92 | 0.83 | 0.97 |
| juv mal SP | 0.96 | 0.47 | 1 |
| juv fem SN | 0.88 | 0.80 | 0.94 |
| juv fem SP | 0.87 | 0.71 | 0.95 |
| ad mal SN | 0.95 | 0.92 | 0.97 |
| ad mal SP | 0.83 | 0.77 | 0.88 |
| ad fem SN | 0.95 | 0.93 | 0.96 |
| ad fem SP | 0.92 | 0.88 | 0.95 |
| **E2 MYXO** |  |  |  |
| juv mal SN | 0.94 | 0.80 | 0.99 |
| juv mal SP | 0.78 | 0.61 | 0.89 |
| juv fem SN | 0.98 | 0.51 | 1 |
| juv fem SP | 0.65 | 0.47 | 0.80 |
| ad mal SN | 0.89 | 0.84 | 0.92 |
| ad mal SP | 0.95 | 0.87 | 0.98 |
| ad fem SN | 0.96 | 0.91 | 0.99 |
| ad fem SP | 0.87 | 0.82 | 0.91 |
| **E2 RHD** |  |  |  |
| juv SN | 0.92 | 0.86 | 0.95 |
| juv SP | 0.74 | 0.59 | 0.84 |
| ad SN | 0.92 | 0.89 | 0.94 |
| ad SP | 0.94 | 0.90 | 0.97 |
| **E3 MYXO** |  |  |  |
| SN | 0.86 | 0.83 | 0.89 |
| SP | 0.94 | 0.92 | 0.96 |
| **E3 RHD** |  |  |  |
| juv SN | 0.88 | 0.82 | 0.92 |
| juv SP | 0.81 | 0.68 | 0.90 |
| ad SN | 0.92 | 0.89 | 0.95 |
| ad SP | 0.96 | 0.83 | 0.99 |

Notation: juv, Juveniles; ad, Adults; fem, Females; mal, Males; SN, Seronegatives; SP, Seropositives.
