# Supplementary material for: Multi-event capture–recapture modeling of host–pathogen dynamics among European rabbit populations exposed to myxoma and Rabbit Hemorrhagic Disease Viruses: common and heterogeneous patterns
Source: Vet Res. 2014 Apr 5;45(1):39. doi: 10.1186/1297-9716-45-39 (PMC4021418; doi:10.1186/1297-9716-45-39)
Supplement: Additional file 4 — Seroconversion estimates with 95% CI for each enclosure (E1, E2 and E3). Seroconversion estimates in the three enclosures as depending on the main effects found through model selection. No age effect was tested because juveniles remain juveniles only for a short time and no recapture from one individual in juvenile state exists. [file 1297-9716-45-39-S4.doc]

| **Main effects** | **Estimates** | **Low 95% CI** | **High 95% CI** |
| --- | --- | --- | --- |
| **E1 MYXO** |  |  |  |
| mal SN SP | 0.39 | 0.27 | 0.54 |
| mal SP SN | 0.07 | 0.03 | 0.17 |
| fem SN SP | 0.22 | 0.15 | 0.32 |
| fem SP SN | 0 | 0 | 0 |
| **E1 RHD** |  |  |  |
| SN SP | 0.28 | 0.23 | 0.35 |
| SP SN | 0.13 | 0.09 | 0.18 |
| **E2 MYXO** |  |  |  |
| mal SN SP | 0.04 | 0.01 | 0.24 |
| mal SP SN | 0.08 | 0.01 | 0.42 |
| fem SN SP | 0.31 | 0.18 | 0.47 |
| fem SP SN | 0.03 | 0.01 | 0.14 |
| **E2 RHD** |  |  |  |
| mal SN SP | 0.14 | 0.07 | 0.29 |
| mal SP SN | 0.15 | 0.04 | 0.41 |
| fem SN SP | 0.44 | 0.28 | 0.61 |
| fem SP SN | 0.18 | 0.08 | 0.34 |
| **E3 MYXO** |  |  |  |
| SN SP | 0.20 | 0.12 | 0.30 |
| SP SN | 0 | 0 | 0 |
| **E3 RHD** |  |  |  |
| mal SN SP | 0.13 | 0.10 | 0.18 |
| mal SP SN | 0.68 | 0.54 | 0.79 |
| fem SN SP | 0.14 | 0.09 | 0.20 |
| fem SP SN | 0.73 | 0.62 | 0.82 |

Notation: as from Additional file 3.
